# Supplementary material for: The Competitive Endogenous RNA (ceRNA) Regulation in Porcine Alveolar Macrophages (3D4/21) Infected by Swine Influenza Virus (H1N1 and H3N2)
Source: Int J Mol Sci. 2022 Feb 7;23(3):1875. doi: 10.3390/ijms23031875 (PMC8836399; doi:10.3390/ijms23031875)
Supplement: Supplementary file 1 [file ijms-23-01875-s001.zip › ijms-1458755-supplementary.pdf]

Supplementary Meterials

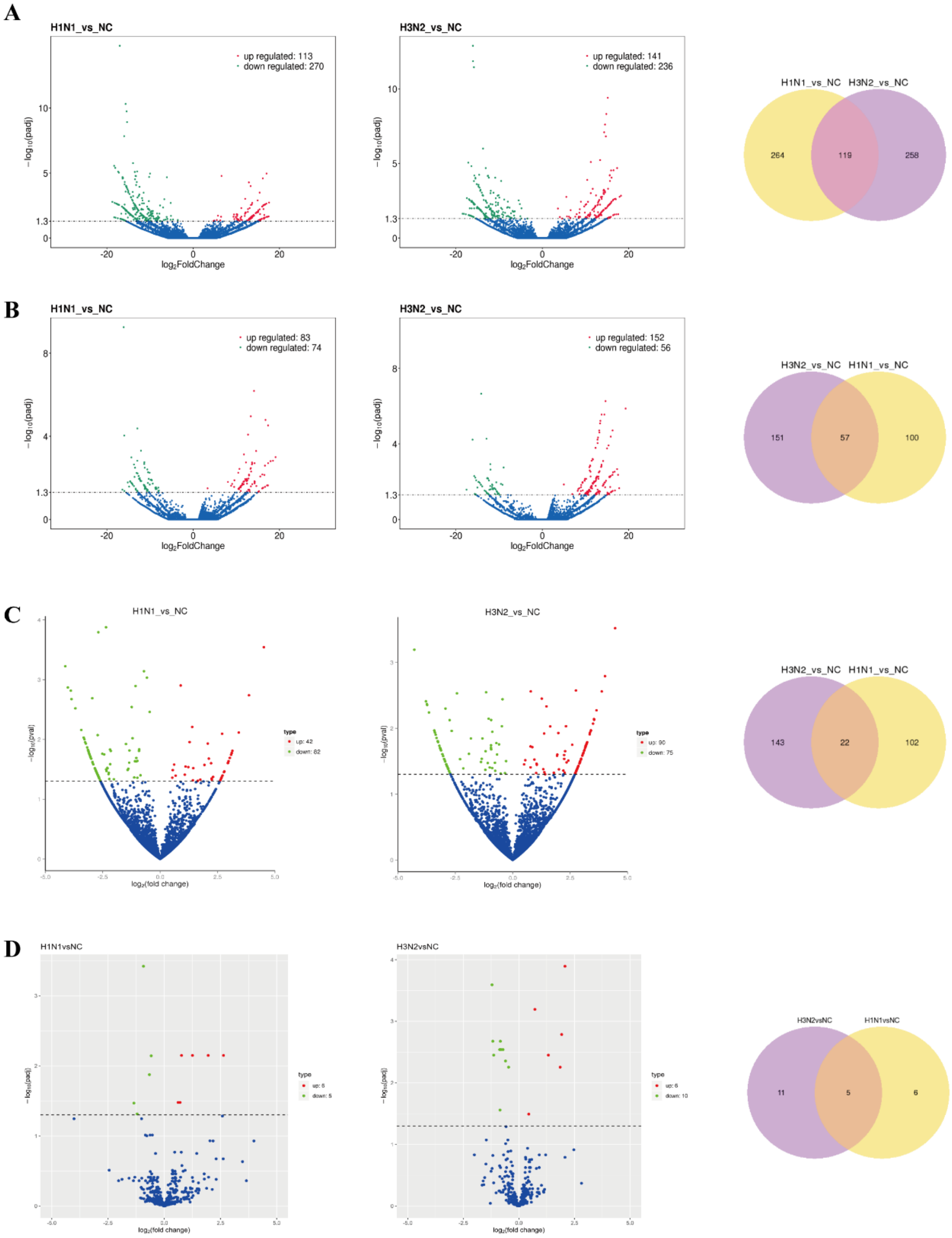

Figure S1. Differential expressed RNA in 3D4/21 cells with/with out H1N1 and H3N2 infection. A-D represent the volcano map and Venn diagram of differentially expressed mRNA, lncRNA, circRNA and miRNA, respectively.

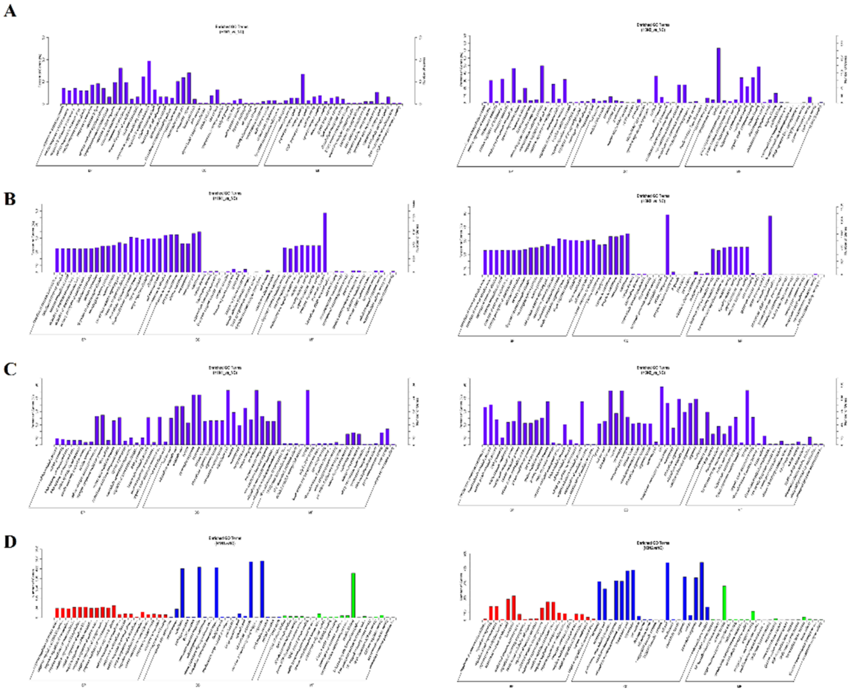

Figure S2. GO enrichment analysis of differentially expressed RNA in 3D4/21 cells infected by H1N1 and H3N2. A represents GO enrichment of differential mRNA. B represents the GO enrichment results of differential lncRNA target genes. C represents the GO enrichment results of differential circRNA source genes. D represents the GO enrichment results of differential miRNA target genes.

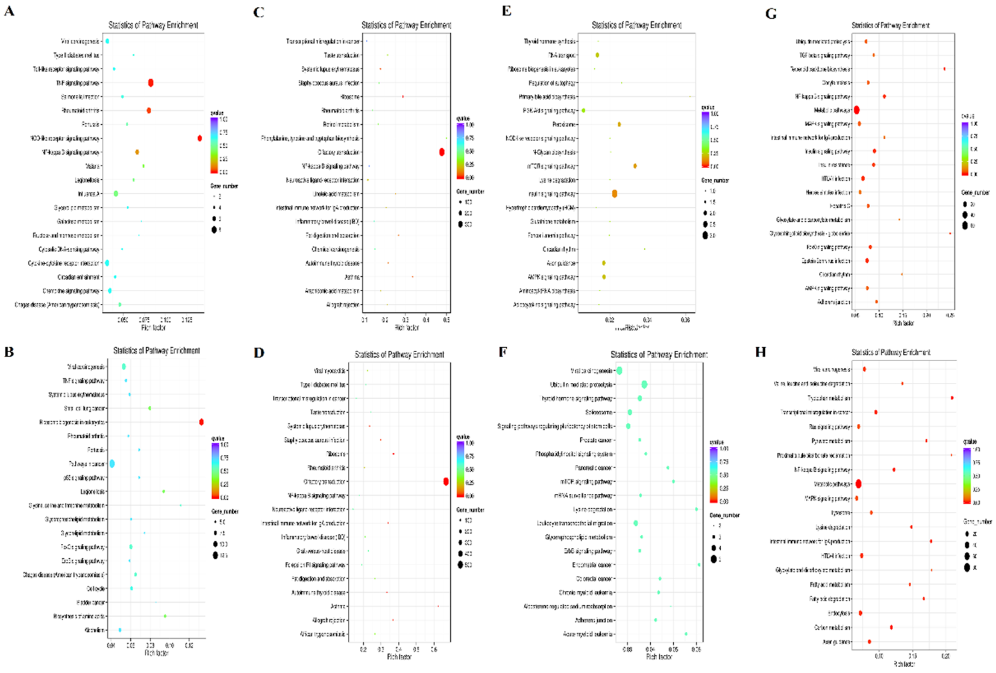

Figure S3. KEGG enrichment analysis of differentially expressed RNA in 3D4/21 cells infected by H1N1 and H3N2. A and B represents KEGG enrichment of differential mRNA. C and D represents the KEGG enrichment results of differential lncRNA target genes. E and F represents the KEGG enrichment results of differential circRNA source genes. G and H represents the KEGG enrichment results of differential miRNA target genes.

Figure S4. Sequencing results of TCONS_00166432-overexpression vector. The sequence in the red box represents the 5' and 3' sequences of the inserted fragment.

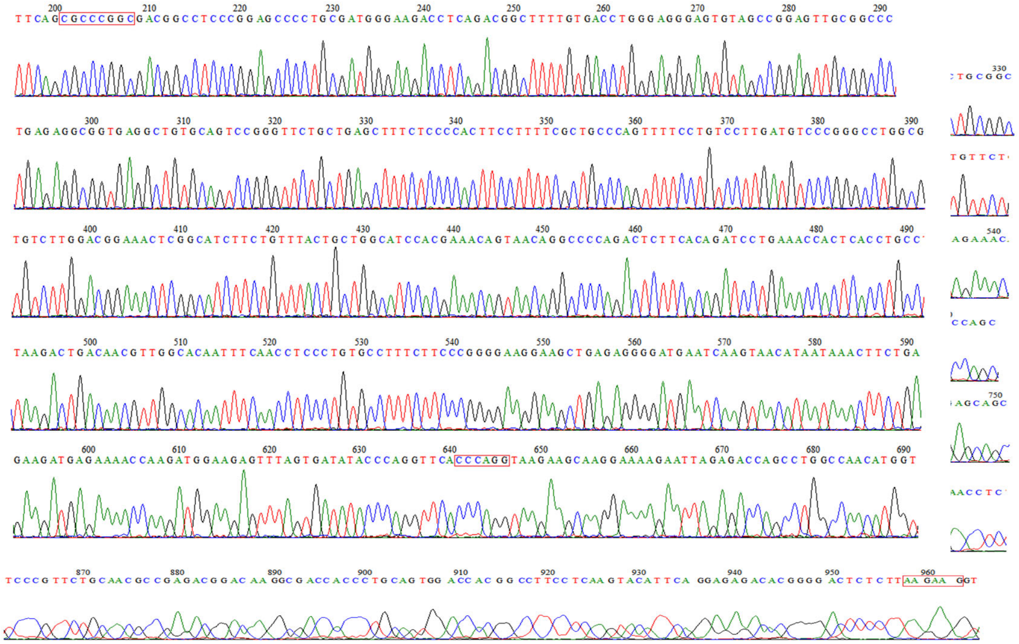

Figure S5. Sequencing results of novel_circ_0004733-overexpression vector. The sequence in the red box represents the 5' and 3' sequences of the inserted fragment.

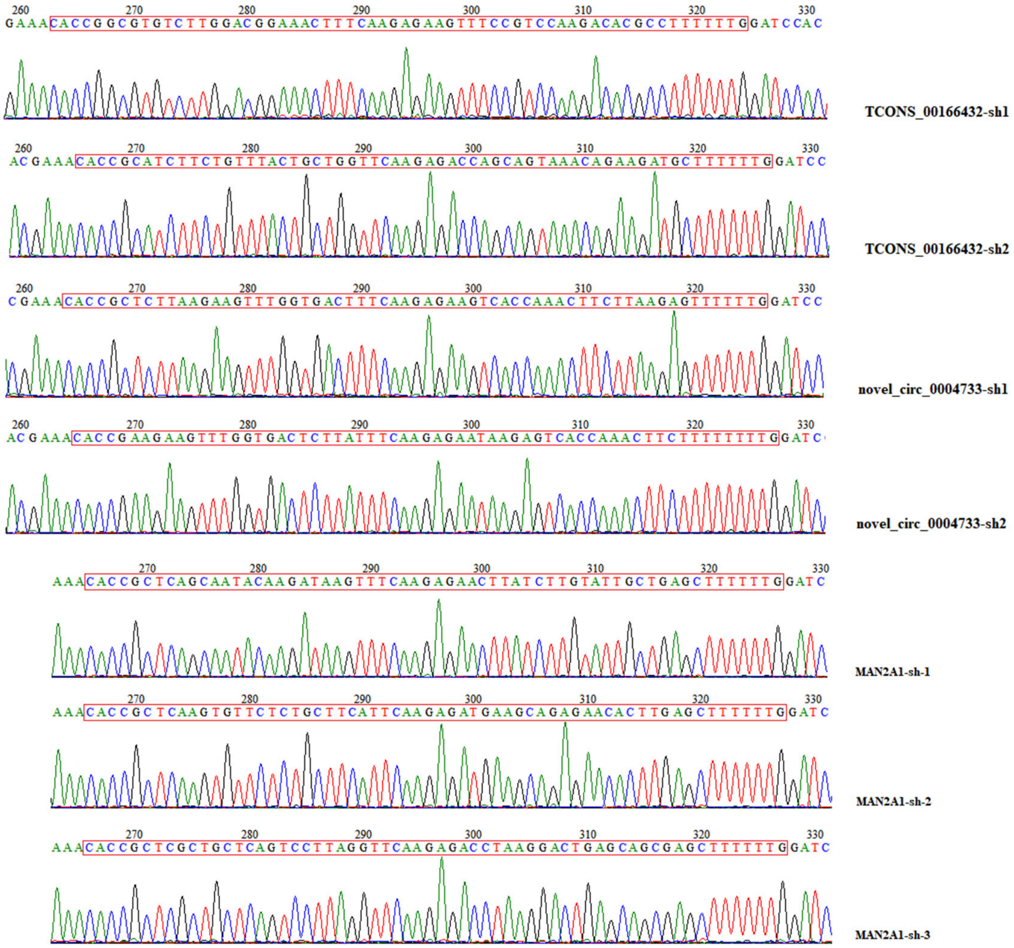

Figure S6. Sequencing results of shRNA vector. The sequence in the red box represents the inserted shRNA sequence.

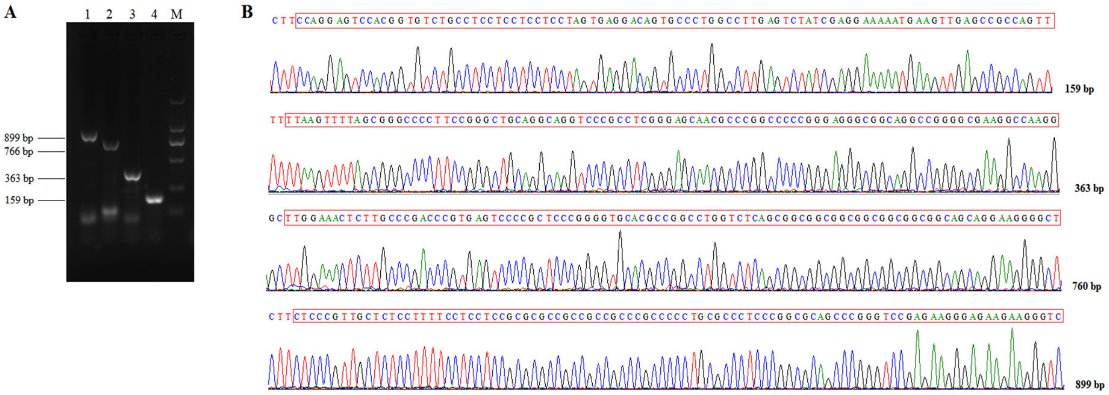

Figure S7. PCR amplification of different segments of *MAN2A1* gene promoter and construction of firefly luciferase vector. A represents the results of agarose gel electrophoresis of the amplified products of different segments of the *MAN2A1* gene promoter. M represents D2000 marker. 1-4 represents MAN2A1-899, MAN2A1-760, MAN2A1-363 and MAN2A1-159, respectively. B represents the sequencing result of the recombinant bacterial solution, and the sequence in the red box represents the 5'sequence of the inserted fragment.

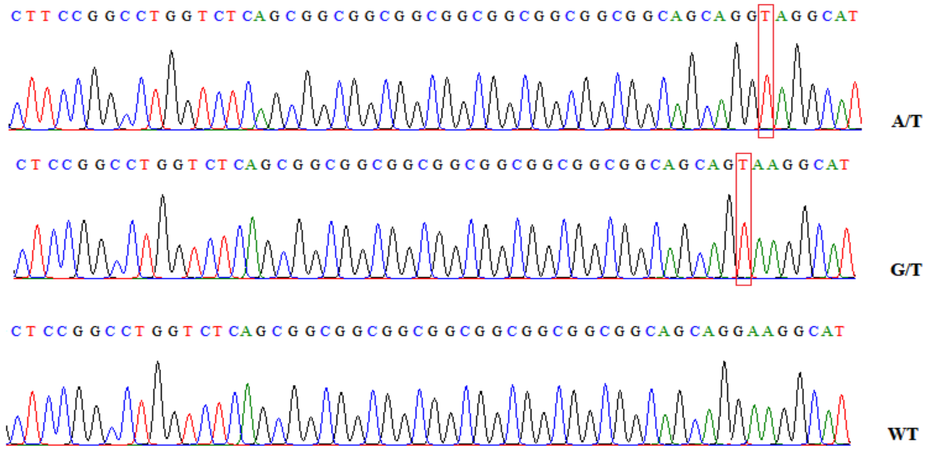

Figure S8. Vector construction of SNP of *MAN2A1* gene promoter.

Table S1 The primer information of qPCR

Name	Sequence of the primer	Length of products (bp)
NP	F: 5'-CCACAAGAGGGGTCCAGATT-3'<br>R: 5'-GGAGATTTCGCTGCACTGAG-3'	180
M	F: 5'-GTGCCGTCGGATGGTAGT-3'<br>R: 5'-CAGTGATGAACCGCAGGAT-3'	196
RIG-I	F: 5'-AAGGACGAAAGGGGAAGGTT-3'<br>R: 5'-AGACGTTGCTCCAGAAATGC-3'	128
NLRP3	F: 5'-GCGTCTCATCAAGGAACACC-3'<br>R: 5'-CTGCTGCTCCCTGGAATACT-3'	179
TLR7	F: 5'-AACTCTGCCCTGTGATGTCA-3'<br>R: 5'-TCCAGGAATCGCTGTCAAGT-3'	179
GAPDH	F: 5'-ACATCATCCCTGCTTCTACTGG-3'<br>R: 5'-CTCGGACGCCTGCTTCAC-3'	188
MAN2A1	F: 5'-GCCAGCTCTCATTGTTGCAA-3'<br>R: 5'-ACCATCCTCCACAGACTCAC-3'	122

DNM2	F: 5'-GCCTCTCATTCTGCAGCTCA-3'<br>R: 5'-GATGCCTTTATTGGTCCCCG-3'	145
SLC27A1	F: 5'-TGTGGTGGCTATCTTCCTGG-3'<br>R: 5'-AATATTGAGCAGAGCGGCCT-3'	94
HSPB8	F: 5'-AAAGTGTGTGTCAACGTGCA-3'<br>R: 5'-CTTCTCCTCGTGTTTGCCAG-3'	96
TBC1D10C	F: 5'-AAAGATGCAGTGCCGGAAAG-3'<br>R: 5'-GTGCCAGGGTTGTTCTTCTG-3'	99
NUBPL	F: 5'-GTTGTGGCTTCTGGAAAGGG-3'<br>R: 5'-AAGGGCCATACACATCCACA-3'	124
TCONS_00166432	F: 5'-CGGAAACTCGGCATCTTCTG-3'<br>R: 5'-GTGCCAACGTTGTCAGTCTT-3'	112
TCONS_00087413	F: 5'-AGGCAGAAAAGAAGAATCGGG-3'<br>R: 5'-AAAGCCTCAGGACCAAGACA-3'	80
novel_circ_0004733	F: 5'-ACATTCAGGAGAGACACGGG-3'<br>R: 5'-CACCAGTGTTGTGCATTTGT-3'	117
novel_circ_0006303	F: 5'-ATGATCTCTCGGGTGCCAAT-3'<br>R: 5'-CGTCTAGTCCGAGCGTGTAA-3'	187
novel_595-RT-F	F: 5'-GTCACCTGGCAGGTACCTCT-3'	-
miR-10391-RT-F	F: 5'-GGAAGGAGACTAACTCCGCC-3'	-
miR-450b-5p-RT-F	F: 5'-GCTTTTGCAATATGTTCCTGAATA-3'	-

Table S2 The oligo sequence of shRNA

Name	Sequence of the oligo (5'→3')
Lnc-sh1F	*CACC*ggcgtgtcttggacggaaactTTCAAGAGAagtttccgtccaagacacgccTTTTTTG
Lnc-sh1R	*GATCC*AAAAAAggcgtgtcttggacggaaactTCTCTTGAAagtttccgtccaagacacgcc
Lnc-sh2F	*CACC*gcatcttctgtttactgctggTTCAAGAGAccagcagtaaacagaagatgcTTTTTTG
Lnc-sh2R	*GATCC*AAAAAAgcatcttctgtttactgctggTCTCTTGAAccagcagtaaacagaagatgc
Circ-sh1F	*CACC*ctcttaagaagtttggtgactTTCAAGAGAagtcaccaaacttcttaagagTTTTTTG
Circ-sh1R	*GATCC*AAAAAActcttaagaagtttggtgactTCTCTTGAAagtcaccaaacttcttaagag
Circ-sh2F	*CACC*aagaagtttggtgactcttatTTCAAGAGAataagagtcaccaaacttcttTTTTTTG
Circ-sh2R	*GATCC*AAAAAAaagaagtttggtgactcttatTCTCTTGAAataagagtcaccaaacttctt
MAN2A1-sh1F	*CACC*gctcagcaatacaagataagtTTCAAGAGAacttatcttgtattgctgagcTTTTTTG
MAN2A1-sh1R	*GATCC*AAAAAAgctcagcaatacaagataagtTCTCTTGAAacttatcttgtattgctgagc
MAN2A1-sh2F	*CACC*gctcaagtgttctctgcttcaTTCAAGAGAtgaagcagagaacacttgagcTTTTTTG
MAN2A1-sh2R	*GATCC*AAAAAAgctcaagtgttctctgcttcaTCTCTTGAAtgaagcagagaacacttgagc
MAN2A1-sh3F	*CACC*gctcgctgctcagtccttaggTTCAAGAGAcctaaggactgagcagcgagcTTTTTTG

MAN2A1-sh3R	*GATCC*AAAAAAgctcgctgctcagtccttaggTCTCTTGAAcctaaggactgagcagcgagc

Note: The italic on shRNA sequences represents the introduced enzyme loci, the lowercase represents the interference sequence and complementary sequence, and underline represents the loop sequence.

Table S3 Oligo information of target site with wild type and mutant type

Name	Oligo sequence
MAN2A1-WT-F	*AGCTT*CATGTACTGTAAGTTTCCTTCCTCGTTTTGGAGGGATAAACAACTTTT*ACGCG*
MAN2A1-WT-R	TAAAAGTTGTTTATCCCTCCAAAACGAGGAAGGAAACTTACAGTACATGA
MAN2A1-mut-F	*AGCTT*CATGTACTGTAAGTTGACGATCGCGTTTTGGAGGGATAAACAACTTTT*ACGCG*
MAN2A1-mut-R	TAAAAGTTGTTTATCCCTCCAAAACGCGATCGTCAACTTACAGTACATGA
circRNA-WT-F	*AGCTT*ATCCGTCCGAATTAATGAACGTTCCTCTTCATCAGCAACAAAACAAAT*ACGCG*
circRNA-WT-R	TATTTGTTTTGTTGCTGATGAAGAGGAACGTTCATTAATTCGGACGGATA
circRNA-mut-F	*AGCTT*ATCCGTCCGAATTAATGAACGCTAGGCTTCATCAGCAACAAAACAAAT*ACGCG*
circRNA-mut-R	TATTTGTTTTGTTGCTGATGAAGCCTAGCGTTCATTAATTCGGACGGATA
lncRNA-WT-F	*AGCTT*AGTCCGGGTTCTGCTGAGCTTTCTCCCCACTTCCTTTTCGCTGCCCAG*ACGCG*
lncRNA-WT-R	TCTGGGCAGCGAAAAGGAAGTGGGGAGAAAGCTCAGCAGAACCCGGACTA
lncRNAo-mut-F	*AGCTT*AGTCCGGGTTCTGCTGAGCTTTCTCCCCAAGCATGCTTCGCTGCCCAG*ACGCG*
lncRNA-mut-R	TCTGGGCAGCGAAGCATGCATGGGGAGAAAGCTCAGCAGAACCCGGACTA

Note: The italic letters represent the introduced enzyme loci of *MIuI* and *Hind* III, the underline letters represent wild type and mutant sequence.

Table S4 Amplification and SNP primers of different promoter regions of *MAN2A1* gene

Name	Sequence of the primer (5'-3)	Length of products (bp)
MAN2A1-F1	CCCA*AGCTT*CTCCCGTTGCTCTCCTTTTC	899
MAN2A1-F2	CCCA*AGCTT*GGAAACTCTTGCCCGACCC	760
MAN2A1-F3	CCCA*AGCTT*TTAAGTTTTAGCGGGCCCCT	363
MAN2A1-F4	CCCA*AGCTT*CTTCCAGGAGTCCACGGTG	159
MAN2A1-R	CATGC*CATGG*CGGTCCAGCATCAGGTAGAG	-
MAN2A1-SNP-F	GGAAACTCTTGCCCGACCC	95
MAN2A1-SNP-R	GGACTGAGCCCCTTCCTG	

Note: F1-F4 represent amplification primers with different regions of the promoter region of *MAN2A1*gene, respectively. R stands for common downstream primer. Italic letters represent the restriction enzymes *Hind*III and *Nco*I.

Table S5 Oligo information in the promoter region SNP sites of *MAN2A1* gene

Name	Sequence of oligo
MAN2A1-WT-F	*AGCTT*CCGGCCTGGTCTCAGCGGCGGCGGCG<br>GCGGCGGCGGCGGCAGCAGGAAGG
MAN2A1-WT-R	*CATGG*CCTTCCTGCTGCCGCCGCCGCCGCCGC<br>CGCCGCCGCTGAGACCAGGCCGG
MAN2A1-G/T-F	*AGCTT*CCGGCCTGGTCTCAGCGGCGGCGGCG<br>GCGGCGGCGGCGGCAGCAGTTAGG
MAN2A1-G/T-R	*CATGG*CCTAACTGCTGCCGCCGCCGCCGCCGC<br>CGCCGCCGCTGAGACCAGGCCGG
MAN2A1-A/T-F	*AGCTT*CCGGCCTGGTCTCAGCGGCGGCGGCG<br>GCGGCGGCGGCGGCAGCAGGTAGG
MAN2A1-A/T-R	*CATGG*CCTACCTGCTGCCGCCGCCGCCGCCGC<br>CGCCGCCGCTGAGACCAGGCCGG

Note: Italic letters in primer F and R represent the restriction enzymes *Hind*III and *Nco*I. The underlined letters represent wild-type and mutation site sequences.

Table S6 Overall statistics for lncRNA sequencing data

Sample ID	Raw Reads	Clean Reads (%)	Q20 (%)	Q30 (%)	GC Content (%)	Total mapped (%)	Accession
H1N1_1	121601720	120504004 (99.10)	97.66	93.16	43.47	62.94	SRR11810843
H1N1_2	166905250	165334148 (99.06)	97.45	92.64	43.97	68.47	SRR11810842
H1N1_3	146030678	144616150 (99.04)	97.75	93.36	43.94	71.84	SRR11810839
H1N1_4	158436640	157216520 (99.23)	97.74	93.49	45.26	71.59	SRR11810838
H3N2_1	139098108	137991624 (99.20)	97.67	93.31	45.99	79.63	SRR11810837
H3N2_2	113184726	111950496 (98.91)	97.54	92.98	47.24	77.5	SRR11810836
H3N2_3	166664822	165211936 (99.13)	97.6	93.11	45.81	71.94	SRR11810835
H3N2_4	182473156	180917310 (99.15)	97.6	93.18	47.7	79.61	SRR11810834
NC_1	145324490	144546820 (99.46)	97.35	92.64	47.91	81.69	SRR11810833
NC_2	149207694	147849784 (99.09)	97.53	93.02	48.32	84.33	SRR11810832
NC_3	135699968	134467098 (99.09)	97.51	92.94	46.89	76.21	SRR11810841
NC_4	156797796	155492816 (99.17)	97.67	93.23	45.95	75.54	SRR11810840

Table S7 Overall statistics for miRNA sequencing data

Sample ID	Raw Reads	Clean Reads (%)	Q20 (%)	Q30 (%)	GC Content (%)	Total mapped (%)	Accession
H1N1_1	18427074	17870889 (96.98)	99.27	97.1	46.65	70.78	SRR11805274
H1N1_2	18484390	18218599 (98.56)	99.17	97.09	46.7	81.72	SRR11805273
H1N1_3	21512324	21238870 (98.73)	99.38	97.38	47.65	81.62	SRR11805270
H1N1_4	21074712	20789173 (98.65)	99.42	97.61	46.81	82.64	SRR11805269
H3N2_1	19508411	19257440 (98.71)	99.34	97.6	46.92	85.35	SRR11805268
H3N2_2	20433050	20169045 (98.71)	99.17	97.11	47.38	85.11	SRR11805267

H3N2_3	17650898	17409715 (98.63)	99.17	97.13	46.75	84.51	SRR11805266
H3N2_4	20344762	20071565 (98.66)	99.42	97.68	47.51	88.42	SRR11805265
NC_1	17466648	17243636 (98.72)	99.15	97.04	46.77	89.01	SRR11805264
NC_2	16048197	15845767 (98.74)	99.17	97.13	46.97	90.79	SRR11805263
NC_3	20140506	19951159 (99.06)	99.2	97.18	46.9	87.36	SRR11805272
NC_4	19208580	18803579 (97.89)	99.36	97.35	47.8	80.92	SRR11805271
